# Supplementary material for: Prevalence of sending, receiving and forwarding sexts among youths: A three-level meta-analysis
Source: PLoS One. 2020 Dec 7;15(12):e0243653. doi: 10.1371/journal.pone.0243653 (PMC7721144; doi:10.1371/journal.pone.0243653)
Supplement: S1 Table — Subject areas excluded: Business, Management and Accounting, Economics, Econometrics and Finance, Biochemistry, Genetics and Molecular Biology and Pharmacology, Toxicology and Pharmaceutics. (DOCX) [file pone.0243653.s002.docx]

| Databases | Published all years to | | Limits applied | Alert activated | |
| --- | --- | --- | --- | --- | --- |
| ERIC  Research field: (“sexting”) in ANY FIELD | February  2020  (in all databases) | | No | No | |
| PsycINFO  Research field: (“sexting”) in ANY FIELD |  |  | No | Weekly  Only new documents | |
| Pubmed  Research field: (“sexting”) in ANY FIELD |  |  | No | No | |
| Scopus  Research field: TITLE-ABS-KEY (sexting) AND  (EXCLUDE (SUBJAREA, "BUSI") OR EXCLUDE (SUBJAREA, "ECON") OR EXCLUDE (SUBJAREA, "BIOC") OR EXCLUDE (SUBJAREA, "PHAR") ) |  |  | Yes * | Weekly  Only new documents | |
| Web of Science  Research field: (“sexting”) in ANY FIELD |  |  | No | Weekly  Only new documents | |
| Gray literature |  | |  |  | |
| Google Scholar  Research field: “sexting”, “sext”, “sexual texting” and “sexual messaging”  Where the words occur: anywhere in the publication | February  2005-2020 | | No | No | |
|  | The authors reviewed the first 100 results of each year since 2005, sorted by relevance | | | | |
| Google  Research field: “sexting”, “sext”, “sexual texting” and “sexual messaging” | February  2020 | No | | | No |
|  | The authors reviewed the first 250 results sorted by relevance | | | | |

**S1 Table. Search strategy used.**

Note. Subject areas excluded: Business, Management and Accounting, Economics, Econometrics and Finance, Biochemistry, Genetics and Molecular Biology and Pharmacology, Toxicology and Pharmaceutics.
