# Supplementary material for: Prevalence of sending, receiving and forwarding sexts among youths: A three-level meta-analysis
Source: PLoS One. 2020 Dec 7;15(12):e0243653. doi: 10.1371/journal.pone.0243653 (PMC7721144; doi:10.1371/journal.pone.0243653)
Supplement: S2 Table — (DOCX) [file pone.0243653.s003.docx]

| Nº | Study | Did not assess sexting prevalence | Prevalence data | Methodological information | Language | Did not meet age criteria |
| --- | --- | --- | --- | --- | --- | --- |
| 1 | Abraham 2015 | - | - | - | - | X |
| 2 | Adam 2019 | - | Not reported | - | - | - |
| 3 | Alonso & Romero 2019 | - | - | - | - | X |
| 4 | APMTV 2009 | - | - | - | - | X |
| 5 | APMTV 2011 | - | - | - | - | X |
| 6 | APMTV 2013 | - | - | - | - | X |
| 7 | Atamari et al. 2017 | X | - | - | - | - |
| 8 | Barrense-Dias et al. 2018 | - | - | - | - | X |
| 9 | Barrense-Dias et al. 2019 | - | - | Insufficient | - | - |
| 10 | Bergmann et al. 2016 | - | - | - | German | - |
| 11 | Boden 2017 | - | - | - | - | X |
| 12 | Boulat et al. 2012 | X | - | - | - | - |
| 13 | Broaddus & Dickson-Gómez 2016 | X | - | - | - | - |
| 14 | Buchanan 2015 | - | - | Measure not stable | - | - |
| 15 | Burén & Lunde 2018 | - | Not calculable | - | - | - |
| 16 | Burić et al. 2018 | - | - | - | Croatian | - |
| 17 | Casas et al. 2019 | - | Not reported | - | - | - |
| 18 | Cheryl 2013 | - | Not calculable | - | - | - |
| 19 | Comartin et al. 2013 | X | - | - | - | - |
| 20 | Cressato 2017 | - | - | - | Italian | - |
| 21 | Dake et al. 2012 | - | - | - | - | X |
| 22 | Dawn 2018 | X | - | - | - | - |
| 23 | Dekker & Koops 2017 | - | - | - | German | - |
| *Continued* | |  |  |  |  |  |

**S2 Table. Excluded studies.**

| Nº | Study | Did not assess sexting prevalence | Prevalence data | Methodological information | Language | Did not meet age criteria |
| --- | --- | --- | --- | --- | --- | --- |
| 24 | Del Rey et al. 2019 | - | Not calculable | - | - | - |
| 25 | Dowdell & Noel 2020 | - | - | - | - | X |
| 26 | Dowdell et al. 2011 | - | - | Measure not stable | - | - |
| 27 | Downs et al. 2013 | - | - | Insufficient | - | - |
| 28 | Drouin & Tobin 2014 | - | - | - | - | X |
| 29 | Drouin et al. 2015 | - | - | - | - | X |
| 30 | Drouin et al. 2017 | - | - | - | - | X |
| 31 | Englander & McCoy 2017 | - | - | Insufficient | - | - |
| 32 | Englander 2012 | - | - | - | - | X |
| 33 | Enyonam 2016 | - | - | - | - | X |
| 34 | Eugene 2015 | - | - | Insufficient | - | - |
| 35 | Evelyn 2018 | - | - | - | - | X |
| 36 | Farber et al. 2012 | X | - | - | - | - |
| 37 | Ferguson 2011 | - | - | - | - | X |
| 38 | Fleschler Peskin et al. 2013 | - | - | - | - | X |
| 39 | Galovan et al. 2018 | - | - | - | - | X |
| 40 | García-Gómez 2019 | - | - | Insufficient | - | - |
| 41 | Gerding & Stevens 2019 | - | Not reported | - | - | - |
| 42 | Giroux 2011 | - | - | - | - | X |
| 43 | Gómez 2019 | - | Not reported | - | - | - |
| 44 | Gómez-Laguna 2018 | X | - | - | - | - |
| 45 | González-Cabrera et al. 2019 | X | - | - | - | - |
| 46 | Guevara-García et al. 2019 | - | - | - | - | X |
| *Continued* | |  |  |  |  |  |

| Nº | Study | Did not assess sexting prevalence | Prevalence data | Methodological information | Language | Did not meet age criteria |
| --- | --- | --- | --- | --- | --- | --- |
| 47 | Hajnalka-Szende 2018 | - | - | - | Romanian | - |
| 48 | Harris et al. 2013 | - | Not calculable | - | - | - |
| 49 | Harrison 2011 | X | - | - | - | - |
| 50 | Hasinoff & Shepherd 2014 | X | - | - | - | - |
| 51 | Hertlein et al. 2015 | X | - | - | - | - |
| 52 | Hinduja & Patchin 2020 | - | Not reported | - | - | - |
| 53 | Hollá 2016 | - | - | - | Slovak | - |
| 54 | Hollá 2017 | - | - | - | Slovak | - |
| 55 | Hua 2012 | X | - | - | - | - |
| 56 | Jonsson et al. 2014 | X | - | - | - | - |
| 57 | Jonsson et al. 2015 | - | - | - | - | X |
| 58 | Kernsmith et al. 2018 | X | - | - | - | - |
| 59 | Kerstens & Stol 2014 | X | - | - | - | - |
| 60 | Kopecký 2012 | - | Repeated | - | - | - |
| 61 | Le 2016 | X | - | - | - | - |
| 62 | Lee et al. 2015 | - | - | - | - | X |
| 63 | Lee et al. 2016 | - | - | - | - | X |
| 64 | Longobardi et al. 2020 | - | Not reported | - | - | - |
| 65 | López Tápia & Martínez Toledo 2018 | - | - | - | - | X |
| 66 | Lucero et al. 2014 | X | - | - | - | - |
| 67 | Marcos-Cuesta 2019 | - | - | - | - | X |
| 68 | Marengo et al. 2019 | - | - | - | - | X |
| 69 | Marganski 2017 | - | - | - | - | X |
| 70 | Mark et al. 2014 | - | - | Insufficient | - | - |
| *Continued* | |  |  |  |  |  |

| Nº | Study | Did not assess sexting prevalence | Prevalence data | Methodological information | Language | Did not meet age criteria |
| --- | --- | --- | --- | --- | --- | --- |
| 71 | Martín-Arias et al. 2013 | X | - | - | - | - |
| 72 | Martínez-Gómez et al. 2018 | - | - | Insufficient | - | - |
| 73 | Marume et al. 2018 | - | - | - | - | X |
| 74 | May 2012 | X | - | - | - | - |
| 75 | McCabe & Johnston 2014 | X | - | - | - | - |
| 76 | McDonald et al. 2018 | - | - | Insufficient | - | - |
| 77 | McMahon 2019 | - | - | - | - | X |
| 78 | Medina & Ruales 2018 | - | - | - | - | X |
| 79 | Migliorato et al. 2018 | - | - | - | Italian | - |
| 80 | Miniguano et al. 2017 | - | - | Insufficient | - | - |
| 81 | Moran et al. 2018 | - | Not reported | - | - | - |
| 82 | Moreira et al. 2019 | X | - | - | - | - |
| 83 | Morelli et al. 2017 | - | - | - | - | X |
| 84 | Murray 2014 | - | - | - | - | X |
| 85 | NCPTUP 2008 | - | - | - | - | X |
| 86 | Ndidi 2018 | - | - | - | - | X |
| 87 | Nguyên & Mark 2014 | X | - | - | - | - |
| 88 | Ochoa 2018 | - | Not calculable | - | - | - |
| 89 | Ojeda et al. 2019 | - | Not reported | - | - | - |
| 90 | Once & Piedra 2018 | - | - | - | - | X |
| 91 | Oswaldo 2012 | - | Not calculable | - | - | - |
| 92 | Paluckaite & Matulaitiene 2017 | - | Not reported | - | - | - |
| 93 | Patchin & Hinduja 2018 | X | - | - | - | - |
| 94 | Patchin & Hinduja 2019 | - | Not calculable | - | - | - |
| *Continued* | |  |  |  |  |  |

| Nº | Study | Did not assess sexting prevalence | Prevalence data | Methodological information | Language | Did not meet age criteria |
| --- | --- | --- | --- | --- | --- | --- |
| 95 | Pellai et al. 2015 | - | - | - | Italian | - |
| 96 | Phippen 2009 | X | - | - | - | - |
| 97 | Pineda et al. 2019 | - | - | Insufficient | - | - |
| 98 | Powell et al. 2019 | - | - | - | - | X |
| 99 | Punina 2018 | - | - | - | - | X |
| 100 | Reed et al. 2016 4 | X | - | - | - | - |
| 101 | Reed et al. 2020 | - | - | - | - | X |
| 102 | Reyns et al. 2013 | - | - | - | - | X |
| 103 | Rial et al. 2018 | - | - | Insufficient | - | - |
| 104 | Ringrose & Harvey 2015 | X | - | - | - | - |
| 105 | Ringrose et al. 2013 | X | - | - | - | - |
| 106 | Rodríguez-Castro et al. 2017 | - | Not reported | - | - | - |
| 107 | Romo et al. 2016 | - | - | Insufficient | - | - |
| 108 | Rood et al. 2015 | - | - | Insufficient | - | - |
| 109 | Rubio-Aurioles et al. 2017 | - | - | Insufficient | - | - |
| 110 | Sánchez-Jimenez et al. 2015 | X | - | - | - | - |
| 111 | Santisteban & Gámez-Guadix 2017 | - | Not reported | - | - | - |
| 112 | Schloms-Madlener 2013 | - | - | - | - | X |
| 113 | Schoeps et al. 2020 | - | Not reported | - | - | - |
| 114 | Seiler 2015 | X | - | - | - | - |
| 115 | Smith-Darden et al. 2017 | X | - | - | - | - |
| 116 | Song et al. 2018 | X | - | - | - | - |
| 117 | Spencer et al. 2015 | - | - | Insufficient | - | - |
| 118 | Speno & Aurbey 2019 | - | Not reported | - | - | - |
| *Continued* | |  |  |  |  |  |

| Nº | Study | Did not assess sexting prevalence | Prevalence data | Methodological information | Language | Did not meet age criteria |
| --- | --- | --- | --- | --- | --- | --- |
| 119 | Stanley et al. 2018 | - | Repeated | - | - | - |
| 120 | Strassberg et al. 2017 | - | - | Insufficient | - | - |
| 121 | Takemoto et al. 2017 | - | - | Insufficient | - | - |
| 122 | Teimouri et al. 2013 | - | Not reported | - | - | - |
| 123 | Temple & Choi 2014 | - | Repeated | - | - | - |
| 124 | Temple et al. 2012 | - | - | - | - | X |
| 125 | Temple et al. 2014 | - | Not reported | - | - | - |
| 126 | Thomson et al. 2018 | - | - | - | - | X |
| 127 | Tomic et al. 2017 | - | Not reported | - | - | - |
| 128 | Tylor et al. 2017 | - | Not reported | - | - | - |
| 129 | Van Oosten & Vandenbosch 2020 | X | - | - | - | - |
| 130 | Van Oosten 2017 | - | - | - | Dutch | - |
| 131 | Van Ouytsel et al. 2016 | X | - | - | - | - |
| 132 | Van Ouytsel et al. 2017 | - | Not calculable | - | - | - |
| 133 | Van Ouytsel et al. 2019 20 | - | Not calculable | - | - | - |
| 134 | Vrselja et al. 2015 | - | - | - | Croatian | - |
| 135 | Wachs et al. 2015 | - | Not reported | - | - | - |
| 136 | Wei & Lo 2013 | X | - | - | - | - |
| 137 | Wei 2012 | X | - | - | - | - |
| 138 | Wolak et al. 2012 | - | Police documents | - | - | - |
| 139 | Wolfe et al. 2013 | - | Repeated | - | - | - |
| 140 | Woolard 2011 | - | - | - | - | X |
| 141 | Wysocki & Childers 2011 | - | - | - | - | X |
| 142 | Yépez-Tito et al. 2018 | - | Not calculable | - | - | - |
| 143 | Zemmels & Khey 2015 | - | - | - | - | X |
| 144 | Zsila et al. 2018 | X | - | - | - | - |
