# Supplementary material for: Prevalence of sending, receiving and forwarding sexts among youths: A three-level meta-analysis
Source: PLoS One. 2020 Dec 7;15(12):e0243653. doi: 10.1371/journal.pone.0243653 (PMC7721144; doi:10.1371/journal.pone.0243653)
Supplement: S3 Table — “HDHD” = attention-deficit/hyperactivity disorder, “na” = Not available, “Yes” = The study provides minimum and maximum age, “Not completely” = Provides at least minimum, maximum or average age, “No” = Does not provide any data. (DOCX) [file pone.0243653.s004.docx]

|  |  |  |  | Study design and sampling  technique | | Quality of the measurement | |  |
| --- | --- | --- | --- | --- | --- | --- | --- | --- |
| Nº | Study | Reference population | Age description | Design | Sampling | Risk of bias | Temporal framework | Response rate |
| 1 | Alfaro-González et al. 2015 | Adolescents | Yes | Cross-sectional | Probabilistic | Insufficient information | Lifetime or undefined | na |
| 2 | Arias Cerón et al. 2018 | Youth | Yes | Cross-sectional | Not probabilistic | Low risk | Last year | na |
| 3 | Baiden et al. 2020 | High school students | Not completely | Cross-sectional | Probabilistic | Significant risk | Lifetime or undefined | na |
| 4 | Baumgartner et al. 2014 | Adolescents | Yes | Cross-sectional | Probabilistic | Significant risk | Last year | na |
| 5 | Beckmeyer et al. 2019 | Adolescents | Yes | Cross-sectional | Probabilistic | Significant risk | Lifetime or undefined | 22.20% |
| 6 | Bermeo 2019 | Adolescents | Yes | Cross-sectional | Not probabilistic | Significant risk | Lifetime or undefined | na |
| 7 | Brinkley et al. 2017 | High school students | Yes | Longitudinal | Not probabilistic | Low risk | Four days | na |
| 8 | Campbell & Park 2014 | Adolescents | Yes | Cross-sectional | Probabilistic | Significant risk | Lifetime or undefined | 11.20% - 13.70% |
| 9 | Chaudhary et al. 2017 | Middle school students | Not completely | Longitudinal | Not probabilistic | Significant risk | Lifetime or undefined | na |
| 10 | Choi et al. 2019 | High school students | Not completely | Longitudinal | Not probabilistic | Low risk | Lifetime or undefined | 62% |
| 11 | Cleary & Najdowski 2019 | Adolescents | Yes | Cross-sectional | Not probabilistic | Significant risk | Last year | na |
| 12 | Cox Communications 2009 | Adolescents | Yes | Cross-sectional | Probabilistic | Insufficient information | Lifetime or undefined | na |
| 13 | Dawson et al. 2019 | Adolescents with HDHD | Yes | Cross-sectional | Not probabilistic | Low risk | Lifetime or undefined | 30.37% |
| *Continued* | |  |  |  |  |  |  |  |

**S3 Table.** **Critical appraisal of the studies**.

| Nº | Study | Reference population | Age description | Design | Sampling | Risk of bias | Temporal framework | Response rate |
| --- | --- | --- | --- | --- | --- | --- | --- | --- |
| 14 | De Graaf et al. 2018 | Youth | Yes | Cross-sectional | Probabilistic | Significant risk | Last six months | na |
| 15 | Dodaj et al. 2019 | High school students | Yes | Longitudinal | Not probabilistic | Low risk | Lifetime or undefined | na |
| 16 | Dolev-Cohen & Ricon 2020 | Middle and high school students | Yes | Cross-sectional | Not probabilistic | Significant risk | Lifetime or undefined | na |
| 17 | Fix et al. 2019 | Adolescents from a correctional | No | Cross-sectional | Not probabilistic | Insufficient information | Lifetime or undefined | na |
| 18 | Frankel et al. 2018 | High school students | No | Cross-sectional | Probabilistic | Significant risk | Last month | 64% |
| 19 | Gámez-Guadix & Mateos-Pérez 2019 | Secondary students | Yes | Longitudinal | Probabilistic | Low risk | Last year | na |
| 20 | Gámez-Guadix & Santisteban 2018 | Secondary students | Yes | Longitudinal | Probabilistic | Significant risk | Last year | na |
| 21 | Gámez-Guadix et al. 2017 | Adolescents | Yes | Cross-sectional | Probabilistic | Low risk | Last year | na |
| 22 | Garitaonandia et al. 2019 | Youths | Yes | Cross-sectional | Probabilistic | Significant risk | Last year | na |
| 23 | Gerding 2016 | High school students | Yes | Cross-sectional | Not probabilistic | Significant risk | Lifetime or undefined | na |
| 24 | Gewirtz-Meydan et al. 2018 | Youth internet users | Yes | Cross-sectional | Probabilistic | Significant risk | Lifetime or undefined | 38.10% |
| 25 | Ghorashi et al. 2019 | High school students | Yes | Cross-sectional | Probabilistic | Significant risk | Last six months | na |
| 26 | Gregg et al. 2018 | High school students | Not completely | Cross-sectional | Not probabilistic | Low risk | Lifetime or undefined | na |
| 27 | Gutiérrez-Gómez 2019 | Adolescents | Yes | Cross-sectional | Not probabilistic | Significant risk | Lifetime or undefined | na |
| 28 | Hinduja & Patchin 2010 | Youth | Yes | Cross-sectional | Probabilistic | Significant risk | Last month | na |
| *Continued* | |  |  |  |  |  |  |  |

| Nº | Study | Reference population | Age description | Design | Sampling | Risk of bias | Temporal framework | Response rate |
| --- | --- | --- | --- | --- | --- | --- | --- | --- |
| 29 | Houck et al. 2014 | Middle school students | Yes | Cross-sectional | Not probabilistic | Significant risk | Last six months | na |
| 30 | Kim et al. 2019 | Adolescents | Yes | Cross-sectional | Probabilistic | Significant risk | Last year | na |
| 31 | Kopecký 2012 | Primary and secondary students | Yes | Cross-sectional | Not probabilistic | Significant risk | Lifetime or undefined | na |
| 32 | Kopecký 2014 | Primary and secondary students | Yes | Cross-sectional | Not probabilistic | Insufficient information | Lifetime or undefined | na |
| 33 | Kopecký 2015 | Pubescent and adolescents | Yes | Cross-sectional | Not probabilistic | Significant risk | Lifetime or undefined | na |
| 34 | Lenhart 2009 | Teens | Yes | Cross-sectional | Probabilistic | Significant risk | Lifetime or undefined | 11.20% - 13.70% |
| 35 | León-Prieto et al. 2017 | High school students | Yes | Cross-sectional | Not probabilistic | Low risk | Lifetime or undefined | na |
| 36 | Lippman & Campbell 2014 | Adolescents | Yes | Cross-sectional | Not probabilistic | Significant risk | Lifetime or undefined | na |
| 37 | Livingstone & Görzig 2014 | Europe multinational | Yes | Cross-sectional | Probabilistic | Significant risk | Last year | 17% - 83% X=42% |
| 38 | Lucić et al. 2020 | Adolescents | Not completely | Cross-sectional | Not probabilistic | Low risk | Last six months | na |
| 39 | Maas et al. 2018 | Female adolescents | Not completely | Cross-sectional | Not probabilistic | Low risk | Lifetime or undefined | na |
| 40 | Maheux et al. 2020 | High school students | Not completely | Cross-sectional | Not probabilistic | Significant risk | Last year | na |
| 41 | Marcum et al. 2014 | High school students | Not completely | Cross-sectional | Not probabilistic | Significant risk | Last year | na |
| 42 | Medina & Verdugo 2018 | Adolescents | Yes | Cross-sectional | Not probabilistic | Low risk | Lifetime or undefined | 45.04% |
| 43 | Mishna et al. 2010 | Middle and high school students | No | Cross-sectional | Probabilistic | Significant risk | Last three months | 17% - 35% |
| *Continued* | |  |  |  |  |  |  |  |

| Nº | Study | Reference population | Age description | Design | Sampling | Risk of bias | Temporal framework | Response rate |
| --- | --- | --- | --- | --- | --- | --- | --- | --- |
| 44 | Mitchell et al. 2012 | Youth | Yes | Cross-sectional | Probabilistic | Significant risk | Last year | na |
| 45 | Molla-Esparza et al. 2020 | Secondary students | Yes | Cross-sectional | Not probabilistic | Significant risk | Lifetime or undefined | na |
| 46 | Montiel et al. 2016 | Secondary students | Yes | Cross-sectional | Probabilistic | Low risk | Last year | na |
| 47 | Naezer 2018 | Youth | Yes | Cross-sectional | Not probabilistic | Insufficient information | Recent experience | na |
| 48 | Nielsen et al. 2015 | Teenage girls | Yes | Cross-sectional | Not probabilistic | Insufficient information | Lifetime or undefined | na |
| 49 | O'Sullivan 2014 | High school students | Yes | Cross-sectional | Not probabilistic | Insufficient information | Lifetime or undefined | na |
| 50 | Patrick et al. 2015 | Secondary students | No | Cross-sectional | Not probabilistic | Significant risk | Lifetime or undefined | na |
| 51 | Quesada et al. 2018 | Adolescents | Yes | Cross-sectional | Not probabilistic | Low risk | Lifetime or undefined | na |
| 52 | Rice et al. 2012 | High school students | Yes | Cross-sectional | Probabilistic | Significant risk | Lifetime or undefined | 76% |
| 53 | Rice et al. 2014 | Middle school students | Yes | Cross-sectional | Probabilistic | Significant risk | Lifetime or undefined | 97.35% |
| 54 | Rice et al. 2018 | High school students | Yes | Cross-sectional | Probabilistic | Significant risk | Lifetime or undefined | 68% |
| 55 | Ricketts et al. 2015 | High school students | Not completely | Cross-sectional | Not probabilistic | Significant risk | Last year | na |
| 56 | Ševčíková 2016 | Europe multinational | Yes | Cross-sectional | Probabilistic | Significant risk | Last year | na |
| 57 | Ševčíková et al. 2018 | Primary and secondary students | Yes | Longitudinal | Not probabilistic | Significant risk | Last six months | na |
| 58 | Soriano et al. 2019 | Adolescents | Yes | Cross-sectional | Probabilistic | Low risk | Lifetime or undefined | na |
| *Continued* | |  |  |  |  |  |  |  |

| Nº | Study | Reference population | Age description | Design | Sampling | Risk of bias | Temporal framework | Response rate |
| --- | --- | --- | --- | --- | --- | --- | --- | --- |
| 59 | Stanley et al. 2018 | Europe multinational | Yes | Cross-sectional | Not probabilistic | Significant risk | Lifetime or undefined | na |
| 60 | Steinberg et al. 2019 | High school students | No | Longitudinal | Not probabilistic | Significant risk | Lifetime or undefined | na |
| 61 | Strassberg et al. 2013 | High school students | No | Cross-sectional | Not probabilistic | Significant risk | Lifetime or undefined | 98% |
| 62 | Strassberg et al. 2014 | High school students | No | Cross-sectional | Not probabilistic | Significant risk | More than a year | na |
| 63 | Strassberg et al. 2017 | High school students | No | Cross-sectional | Not probabilistic | Significant risk | Lifetime or undefined | 95% |
| 64 | Titchen et al. 2019 | Young Adolescents | Yes | Cross-sectional | Not probabilistic | Significant risk | Lifetime or undefined | 93.93% |
| 65 | V. Ouytsel et al. 2019a | Early Adolescents | Yes | Cross-sectional | Not probabilistic | Significant risk | Last six months | na |
| 66 | V. Ouytsel et al. 2019b | Secondary students | Yes | Cross-sectional | Not probabilistic | Significant risk | Last six months | na |
| 67 | V. Ouytsel, Ponnet et al. 2014 | Secondary students | Yes | Cross-sectional | Not probabilistic | Significant risk | Last six months and Lifetime | na |
| 68 | V. Ouytsel, Van Gool et al. 2014 | Secondary students | Yes | Cross-sectional | Not probabilistic | Significant risk | Unclear | na |
| 69 | Vanden Abeele et al. 2012 | High school students | Not completely | Cross-sectional | Not probabilistic | Significant risk | Last two months | na |
| 70 | Velarde 2014 | High school students | No | Cross-sectional | Not probabilistic | Significant risk | Lifetime or undefined | na |
| 71 | Villacampa 2016 | Secondary students | Yes | Cross-sectional | Not probabilistic | Significant risk | Lifetime or undefined | na |
| 72 | Villanueva & Serrano 2019 | Adolescents | Yes | Cross-sectional | Not probabilistic | Significant risk | Last month | na |
| 73 | Wachs et al. 2017 | Secondary students | Yes | Cross-sectional | Not probabilistic | Significant risk | Last year | na |
| *Continued* | |  |  |  |  |  |  |  |

| Nº | Study | Reference population | Age description | Design | Sampling | Risk of bias | Temporal framework | Response rate |
| --- | --- | --- | --- | --- | --- | --- | --- | --- |
| 74 | Walrave et al. 2014 | Secondary students | Yes | Cross-sectional | Not probabilistic | Significant risk | Last two months | na |
| 75 | West et al. 2014 | High school students | Yes | Cross-sectional | Not probabilistic | Significant risk | Daily | na |
| 76 | Wolfe et al. 2016 | Teenagers | Yes | Cross-sectional | Probabilistic | Significant risk | Lifetime or undefined | 47% |
| 77 | Wood et al. 2015 | Europe multinational | Yes | Cross-sectional | Not probabilistic | Significant risk | Lifetime or undefined | na |
| 78 | Woodward et al. 2017 | Rural high school students | Not completely | Cross-sectional | Not probabilistic | Significant risk | Lifetime or undefined | 25.70% |
| 79 | Ybarra & Michell 2014 | Adolescents | Yes | Cross-sectional | Probabilistic | Significant risk | Last year | 7% |

Table legend. “HDHD” = attention-deficit/hyperactivity disorder, “na” = Not available, “Yes” = The study provides minimum and maximum age, “Not completely” = Provides at least minimum, maximum or average age, “No” = Does not provide any data.
