# Supplementary material for: Prevalence of sending, receiving and forwarding sexts among youths: A three-level meta-analysis
Source: PLoS One. 2020 Dec 7;15(12):e0243653. doi: 10.1371/journal.pone.0243653 (PMC7721144; doi:10.1371/journal.pone.0243653)
Supplement: S1 Appendix — (DOCX) [file pone.0243653.s006.docx]

**S1 Appendix.** **References included in the meta-analysis.**

1. Alfaro González M, Vázquez Fernández ME, Fierro Urturi A, Herrero Bregón B, Muñoz Moreno MF, Rodríguez Molinero L. Uso y riesgos de las tecnologías de la información y comunicación en adolescentes de 13-18 años. Acta Pediatr Española. 2015;73: e126–e135.

2. Arias Cerón M, Buendía Eisman L, Fernández Palomares F. Grooming, Cyberbullying and Sexting in Chile according of sex and school management or administrative dependency. Rev Chil Pediatría. 2018;89: 352–360. doi:10.4067/S0370-41062018005000201

3. Baiden F, Amankwah J, Owusu A. Sexting among high school students in a metropolis in Ghana: An exploratory and descriptive study. J Child Media. 2020; Advance online publication. doi:10.1080/17482798.2020.1719854

4. Baumgartner SE, Sumter SR, Peter J, Valkenburg PM, Livingstone S. Does country context matter? Investigating the predictors of teen sexting across Europe. Comput Human Behav. 2014;34: 157–164. doi:10.1016/j.chb.2014.01.041

5. Beckmeyer JJ, Herbenick D, Fu TC, Dodge B, Reece M, Fortenberry JD. Characteristics of Adolescent Sexting: Results from the 2015 National Survey of Sexual Health and Behavior. J Sex Marital Ther. 2019;45: 767–780. doi:10.1080/0092623X.2019.1613463

6. Bermeo FI. Factores de riesgo sociodemográficos, familiares y personales asociados al Sexting en estudiantes de dos instituciones educativas de la ciudad de Cuenca. [Unpublished Master’s Thesis]. Universidad de Cuenca. 2019.

7. Brinkley DY, Ackerman RA, Ehrenreich SE, Underwood MK. Sending and receiving text messages with sexual content: Relations with early sexual activity and borderline personality features in late adolescence. Comput Human Behav. 2017;70: 119–130. doi:10.1016/j.chb.2016.12.082

8. Campbell SW, Park YJ. Predictors of mobile sexting among teens: Toward a new explanatory framework. Mob Media Commun. 2014;2: 20–39. doi:10.1177/2050157913502645

9. Chaudhary P, Peskin M, Temple JR, Addy RC, Baumler E, Ross S. Sexting and Mental Health: A School-Based Longitudinal Study among Youth in Texas. J Appl Res Child. 2017;8: Article 11. Available: http://digitalcommons.library.tmc.edu/childrenatrisk/vol8/iss1/11

10. Choi HJ, Mori C, Van Ouytsel J, Madigan S, Temple JR. Adolescent Sexting Involvement Over 4 Years and Associations With Sexual Activity. J Adolesc Heal. 2019;65: 738–744. doi:10.1016/j.jadohealth.2019.04.026

11. Cleary HMD, Najdowski CJ. Awareness of Sex Offender Registration Policies and Self-Reported Sexual Offending in a Community Sample of Adolescents. Sex Res Soc Policy. 2019; Advance online publication. doi:10.1007/s13178-019-00410-3

12. Communications C. Teen online & wireless safety survey: cyberbullying, sexting, and parental controls. 2009. Available: http://ksdresources.pbworks.com/f/2009_teen_survey_internet_and_wireless_safety%5B1%5D.pdf

13. Dawson AE, Wymbs BT, Evans SW, DuPaul GJ. Exploring how adolescents with ADHD use and interact with technology. J Adolesc. 2019;71: 119–137. doi:10.1016/j.adolescence.2019.01.004

14. De Graaf H, Verbeek M, Van den Borne M, Meijer S. Offline and Online Sexual Risk Behavior among Youth in the Netherlands: Findings from “Sex under the Age of 25”. Front public Heal. 2018;6: 1–10. doi:10.3389/fpubh.2018.00072

15. Dodaj A, Sesar K, Jerinić S. A Prospective Study of High-School Adolescent Sexting Behavior and Psychological Distress. J Psychol Interdiscip Appl. 2020;154: 111–128. doi:10.1080/00223980.2019.1666788

16. Dolev-Cohen M, Ricon T. Demystifying sexting: Adolescent sexting and its associations with parenting styles and sense of parental social control in Israel. Cyberpsychology. 2020;14: Article 6. doi:10.5817/CP2020-1-6

17. Fix RL, Falligant JM, Alexander AA, Burkhart BR. Race and Victim Age Matter: Sexual Behaviors and Experiences Among Confined African American and European American Youth With Sexual and Nonsexual Offenses. Sex Abus. 2019;31: 50–72. doi:10.1177/1079063217720926

18. Frankel AS, Bass SB, Patterson F, Dai T, Brown D. Sexting, Risk Behavior, and Mental Health in Adolescents: An Examination of 2015 Pennsylvania Youth Risk Behavior Survey Data. J Sch Health. 2018;88: 190–199. doi:10.1111/josh.12596

19. Gámez-Guadix M, de Santisteban P. “Sex Pics?”: Longitudinal Predictors of Sexting Among Adolescents. J Adolesc Heal. 2018;63: 608–614. doi:10.1016/j.jadohealth.2018.05.032

20. Gámez-Guadix M, Mateos-Pérez E. Longitudinal and reciprocal relationships between sexting, online sexual solicitations, and cyberbullying among minors. Comput Human Behav. 2019;94: 70–76. doi:10.1016/j.chb.2019.01.004

21. Gámez-Guadix M, de Santisteban P, Resett S. Sexting among Spanish adolescents: Prevalence and personality profiles. Psicothema. 2017;29: 29–34. doi:10.7334/psicothema2016.222

22. Garitaonandia C, Karrera I, Larranaga N. Media convergence, risk and harm to children online. Doxa Comun. 2019; 179–199. doi:10.31921/doxacom.n28a10

23. Gerding A. Adolescent sexting: An examination of the psychosocial contributions to the creation and sharing of sexual images. (Publication No. AAI10628994) [Doctoral Thesis, University of Missouri - Columbia] ProQuest Dissertations and Theses Global. 2016.

24. Gewirtz-Meydan A, Mitchell KJ, Rothman EE. What do kids think about sexting? Comput Human Behav. 2018;86: 256–265. doi:10.1016/j.chb.2018.04.007

25. Ghorashi Z, Loripoor M, Lotfipur-Rafsanjani SM. Mobile access and sexting prevalence in high school students in rafsanjan city, Iran in 2015. Iran J Psychiatry Clin Psychol. 2019;24: 416–425. doi:10.32598/ijpcp.24.4.416

26. Gregg D, Somers CL, Pernice FM, Hillman SB, Kernsmith P. Sexting Rates and Predictors From an Urban Midwest High School. J Sch Health. 2018;88: 423–433. doi:10.1111/josh.12628

27. Gutiérrez Gómez I. Estudio estadístico que muestra la presencia de las conductas (ciberbullying, sexting y cibergrooming) a través de la red social facebook, en los alumnos de la Escuela Secundaria Técnica n°1 “Andrés Álvaro García.” [Bachelor’s Thesis, Universidad Autónoma del Estado de México] Repositorio Institucional de la Universidad Autónoma del Estado de México. 2019.

28. Hinduja S, Patchin JW. Sexting. A brief guide for educators and parents. Cyberbullying Research Center.; 2010. Available: https://cyberbullying.org/sexting-a-brief-guide-for-educators-and-parents

29. Houck CD, Barker D, Rizzo C, Hancock E, Norton A, Brown LK. Sexting and sexual behavior in at-risk adolescents. Pediatrics. 2014;133: e276–e282. doi:10.1542/peds.2013-1157

30. Kim S, Martin-Storey A, Drossos A, Barbosa S, Georgiades K. Prevalence and Correlates of Sexting Behaviors in a Provincially Representative Sample of Adolescents. Can J Psychiatry. 2019; Advance Online Publication. doi:10.1177/0706743719895205

31. Kopecky K. Sexting among Czech Preadolescents and Adolescents. New Educ Rev. 2012;28: 39–48.

32. Kopecky K. Cyberbullying and Sexting between Children and Adolescents - Comparative Study. In: Sandu A, Caras A, editors. LUMEN 2014 - From theory to Inquiry in Social Sciences. Romania: Elsevier Ltd; 2014. pp. 467–471. doi:10.1016/j.sbspro.2014.08.292

33. Kopecky K. Sexting among Slovak Pubescents and Adolescent Children. In: Vasile C, Singer F., Stan E, editors. Internacional Conference EPC-TKS 2015. SARA BURGERHARTSTRAAT 25, PO BOX 211, 1000 AE AMSTERDAM, NETHERLANDS: Elsevier Ltd; 2015. pp. 244–250. doi:10.1016/j.sbspro.2015.08.289

34. Lenhart A. Teens and Sexting: How and why minor teens are sending sexually suggestive nude or nearly nude images via text messaging. Washington, DC; 2009. Available: http://www.ncdsv.org/images/pewinternet_teensandsexting_12-2009.pdf

35. León Prieto ME, Vargas Romero JC, Guillén Torres IG. El nivel de incidencia del sexting en adolescentes de 1ero a 3ero de bachillerato general unificado de la unidad educativa particular “Santo Domingo de Guzmán” en el periodo 2016-2017. Rev Electrónica Psicol Iztacala. 2017;20: 165–181. Available: http://www.revistas.unam.mx/index.php/repi/article/view/61789

36. Lippman JR, Campbell SW. Damned if you do, damned if you don’t…if you’re a girl: Relational and normative contexts of adolescent sexting in the United States. J Child Media. 2014;8: 371–386. doi:10.1080/17482798.2014.923009

37. Livingstone S, Görzig A. When adolescents receive sexual messages on the internet: Explaining experiences of risk and harm. Comput Human Behav. 2014;33: 8–15. doi:10.1016/j.chb.2013.12.021

38. Lucić M, Baćak V, Štulhofer A. The role of peer networks in adolescent pornography use and sexting in Croatia. J Child Media. 2020;14: 110–127. doi:10.1080/17482798.2019.1637356

39. Maas M, Bray B, Noll J. A Latent Class Analysis of Online Sexual Experiences and Offline Sexual Behaviors Among Female Adolescents. J Res Adolesc. 2018;28: 731–747. doi:10.1111/jora.12364

40. Maheux AJ, Evans R, Widman L, Nesi J, Prinstein MJ, Choukas-Bradley S. Popular peer norms and adolescent sexting behavior. J Adolesc. 2020;78: 62–66. doi:10.1016/j.adolescence.2019.12.002

41. Marcum CD, Higgins GE, Ricketts ML. Sexting behaviors among adolescents in rural North Carolina: A theoretical examination of low self-control and deviant peer association. Int J Cyber Criminol. 2014;8: 68–78.

42. Medina MO, Verdugo CK. Autoconcepto y sexting en adolescentes de 15 a 18 años en la ciudad de Cuenca. (Publication No. TPSC;84) [Bachelor’s Thesis, Universidad de Cuenca - Ecuador] Respositorio Institutcional de la Universidad de Cuenca. 2018. Available: http://dspace.ucuenca.edu.ec/handle/123456789/30624

43. Mishna F, Cook C, Gadalla T, Daciuk J, Solomon S. Cyber bullying behaviors among middle and high school students. Am J Orthopsychiatry. 2010;80: 362–374. doi:10.1111/j.1939-0025.2010.01040.x

44. Mitchell KJ, Finkelhor D, Jones LM, Wolak J. Prevalence and Characteristics of Youth Sexting: A National Study. Pediatrics. 2012;129: 13–20. doi:10.1542/peds.2011-1730

45. Molla-Esparza C, López-González E, Losilla JM. Sexting Prevalence and Socio-Demographic Correlates in Spanish Secondary School Students. Sex Res Soc Policy. 2020; Advance online publication. doi:10.1007/s13178-020-00434-0

46. Montiel I, Carbonell E, Pereda N. Multiple online victimization of Spanish adolescents: Results from a community sample. Child Abus Negl. 2016;52: 123–134. doi:10.1016/j.chiabu.2015.12.005

47. Naezer M. From risky behaviour to sexy adventures: reconceptualising young people’s online sexual activities. Cult Heal Sex. 2018;20: 715–729. doi:10.1080/13691058.2017.1372632

48. Nielsen S, Paasonen S, Spisak S. ‘Pervy role-play and such’: girls’ experiences of sexual messaging online. Sex Educ. 2015;15: 472–485. doi:10.1080/14681811.2015.1048852

49. O’Sullivan LF. Linking Online Sexual Activities to Health Outcomes Among Teens. In: Lefkowitz ES, Vasilenko SA, editors. Positive and negative outcomes of sexual behaviors. 989 MARKET STREET, SAN FRANCISCO, CA 94103-1741 USA: Wiley Periodicals, Inc.; 2014. pp. 37–51. doi:10.1002/cad.20059

50. Patrick K, Heywood W, Pitts MK, Mitchell A. Demographic and behavioural correlates of six sexting behaviours among Australian secondary school students. Sex Health. 2015;12: 480–487. doi:10.1071/SH15004

51. Quesada S, Fernández-González L, Calvete E. El sexteo (sexting) en la adolescencia: Frecuencia y asociación con la victimización de ciberacoso y violencia en el noviazgo. Behav Psychol. 2018;26: 225–242.

52. Rice E, Rhoades H, Winetrobe H, Sanchez M, Montoya J, Plant A, et al. Sexually Explicit Cell Phone Messaging Associated With Sexual Risk Among Adolescents. Pediatrics. 2012;130: 667–673. doi:10.1542/peds.2012-0021

53. Rice E, Gibbs J, Winetrobe H, Rhoades H, Plant A, Montoya J, et al. Sexting and sexual behavior among middle school students. Pediatrics. 2014;134: e21–e28. doi:10.1542/peds.2013-2991

54. Rice E, Craddock J, Hemler M, Rusow J, Plant A, Montoya J, et al. Associations between sexting behaviors and sexual behaviors among mobile phone-owning teens in Los Angeles. Child Dev. 2018;89: 110–117. doi:10.1111/cdev.12837

55. Ricketts ML, Maloney C, Marcum CD, Higgins GE. The effect of Internet related problems on the sexting behaviors of juveniles. Am J Crim Justice. 2015;40: 270–284. doi:10.1007/s12103-014-9247-5

56. Ševčíková A, Blinka L, Daneback K. Sexting as a predictor of sexual behavior in a sample of Czech adolescents. Eur J Dev Psychol. 2018;15: 426–437. doi:10.1080/17405629.2017.1295842

57. Ševčíková A. Girls’ and boys’ experience with teen sexting in early and late adolescence. J Adolesc. 2016;51: 156–162. doi: 10.1016/j.adolescence.2016.06.007

58. Soriano Ayala E, Cala VC, Bernal Bravo C. Factores socioculturales y psicológicos en el sexting adolescente: Un estudio transcultural. Rev Educ. 2019;384: 175–197. doi:10.4438/1988-592X-RE-2019-384-407

59. Stanley N, Barter C, Wood M, Aghtaie N, Larkins C, Lanau A, et al. Pornography, Sexual Coercion and Abuse and Sexting in Young People’s Intimate Relationships: A European Study. J Interpers Violence. 2018;33: 2919–2944. doi:10.1177/0886260516633204

60. Steinberg DB, Simon VA, Victor BG, Kernsmith PD, Smith-Darden JP. Onset Trajectories of Sexting and Other Sexual Behaviors Across High School: A Longitudinal Growth Mixture Modeling Approach. Arch Sex Behav. 2019;48: 2321–2331. doi:10.1007/s10508-019-1414-9

61. Strassberg DS, McKinnon RK, Sustaita MA, Rullo J. Sexting by High School Students: An Exploratory and Descriptive Study. Arch Sex Behav. 2013;42: 15–21. doi:10.1007/s10508-012-9969-8

62. Strassberg DS, Rullo JE, Mackaronis JE. The sending and receiving of sexually explicit cell phone photos (“Sexting”) while in high school: One college’s students’ retrospective reports. Comput Human Behav. 2014;41: 177–183. doi:10.1016/j.chb.2014.09.008

63. Strassberg DS, Cann D, Velarde V. Sexting by High School Students. Arch Sex Behav. 2017;46: 1667–1672. doi:10.1007/s10508-016-0926-9

64. Titchen KE, Maslyanskaya S, Silver EJ, Coupey SM. Sexting and Young Adolescents: Associations with Sexual Abuse and Intimate Partner Violence. J Pediatr Adolesc Gynecol. 2019;32: 481–486. doi:10.1016/j.jpag.2019.07.004

65. Van Ouytsel J, Walrave M, Ponnet K. Sexting within adolescents’ romantic relationships: How is it related to perceptions of love and verbal conflict? Comput Human Behav. 2019;97: 216–221. doi:10.1016/j.chb.2019.03.029

66. Van Ouytsel J, Walrave M, Ponnet K. An Exploratory Study of Sexting Behaviors Among Heterosexual and Sexual Minority Early Adolescents. J Adolesc Heal. 2019;65: 621–626. doi:10.1016/j.jadohealth.2019.06.003

67. Van Ouytsel J, Ponnet K, Walrave M. The associations between adolescents’ consumption of pornography and music videos and their sexting behavior. Cyberpsychol Behav Soc Netw. 2014;17: 772–778. doi:10.1089/cyber.2014.0365

68. Van Ouytsel J, Van Gool E, Ponnet K, Walrave M. Brief report: The association between adolescents’ characteristics and engagement in sexting. J Adolesc. 2014;37: 1387–1391. doi: 10.1016/j.adolescence.2014.10.004

69. Vanden Abeele M, Roe K, Eggermont S. An exploration of adolescents’ sexual contact and conduct risks through mobile phone use. Communications. 2012;37: 55–77. doi:10.1515/commun-2012-0003

70. Velarde V. The exchange of sexually explicit cell phone pictures (sexting) among high school students. (Publication No. 205929) [Bachelor’s Thesis, University of Utah - Utah] Digital Library of the University of Utah. 2014. Available: https://collections.lib.utah.edu/details?id=205929

71. Villacampa C. Sexting: prevalencia, características personales y conductuales y efectos en una muestra de adolescentes en España. Rev Gen Derecho Penal. 2016;25: 1–36.

72. Villanueva Blasco VJ, Serrano Bernal S. Patrón de uso de internet y control parental de redes sociales como predictor de sexting en adolescentes: una perspectiva de género. Rev Psicol y Educ. 2019;14: 16–26. doi:10.23923/rpye2019.01.168

73. Wachs S, Wright MF, Wolf KD. Psychological Correlates of Teen Sexting in three Countries - Direct and Indirect Associations between Self-control, Self-esteem, and Sexting. Int J Dev Sci. 2017;11: 109–120. doi:10.3233/DEV-160212

74. Walrave M, Heirman W, Hallam L. Under pressure to sext? Applying the theory of planned behaviour to adolescent sexting. Behav Inf Technol. 2014;33: 86–98. doi:10.1080/0144929X.2013.837099

75. West JH, Lister CE, Hall PC, Crookston BT, Snow PR, Zvietcovich ME, et al. Sexting among peruvian adolescents. BMC Public Health. 2014;14: 811. doi:10.1186/1471-2458-14-811

76. Wolfe SE, Marcum CD, Higgins GE, Ricketts ML. Routine Cell Phone Activity and Exposure to Sext Messages: Extending the Generality of Routine Activity Theory and Exploring the Etiology of a Risky Teenage Behavior. Crime Delinq. 2013;62: 614–644. doi:10.1177/0011128714541192

77. Wood M, Barter C, Stanley N, Aghtaie N, Larkins C. Images across Europe: The sending and receiving of sexual images and associations with interpersonal violence in young people’s relationships. Child Youth Serv Rev. 2015;59: 149–160. doi:10.1016/j.childyouth.2015.11.005

78. Woodward VH, Evans M, Brooks M. Social and Psychological Factors of Rural Youth Sexting: An Examination of Gender-Specific Models. Deviant Behav. 2017;38: 461–476. doi:10.1080/01639625.2016.1197020

79. Ybarra ML, Mitchell KJ. “Sexting” and Its Relation to Sexual Activity and Sexual Risk Behavior in a National Survey of Adolescents. J Adolesc Heal. 2014;55: 757–764. doi:10.1016/j.jadohealth.2014.07.012
