# Supplementary material for: Prevalence of sending, receiving and forwarding sexts among youths: A three-level meta-analysis
Source: PLoS One. 2020 Dec 7;15(12):e0243653. doi: 10.1371/journal.pone.0243653 (PMC7721144; doi:10.1371/journal.pone.0243653)
Supplement: S2 Appendix — (DOCX) [file pone.0243653.s007.docx]

**S2 Appendix. Relevant R code and graphs.**

###########################

### ###

#### INDEX OF CONTENTS ####

### ###

###########################

#- LIBRARIES AND CONSTANTS

#--- Packages

#--- Libraries

#--- Constants

#- READING DATA

#--- Read Wide Format Data

#--- Transform Data to Long Format

#--- Add Effect Sizes to Data

#- PREVALENCES ESTIMATION

#--- Joint Model Fit for All Three Sexting Experiences

#--- Proportion Estimates, Confidence Intervals and Credibility/Probability Intervals

#- MODERATOR ANALYSIS

#--- Document Type as an Example of Categorical Moderator

#--- Year of data collection as an Example of Quantitative Moderator

#- DIAGNOSTIC FUNCTIONS AND GRAPHS

#--- Profile Likehood plots

#--- Analysis of Potential Influential Studies or Observations

#--- Analysis of a Potential Publication Bias

#- FOREST PLOT

###############################

## ##

### LIBRARIES AND CONSTANTS ###

## ##

###############################

#Packages

#--------

install.packages("metafor")

install.packages("tidyr")

#Libraries

#---------

library(metafor)

library(tidyr)

#Constants

#---------

route = "C:/Users/Documents/R/Projects/"

dec = 2

meth = "REML"

####################

## ##

### READING DATA ###

## ##

####################

#Read Wide Format Data (Multiple study outcomes in the same data row)

#--------------------------------------------------------------------

datSext <- read.csv2(paste(rute, "Sexting.csv", sep = ""), header = TRUE, na.strings = "NA", dec = ".")

#Transform Data to Long Format (Each outcome in a separate row)

#--------------------------------------------------------------

datSextLong <- gather(datSext, Action, ESize, n_SENDING, n_RECEIV, n_FORW, factor_key = TRUE, na.rm = TRUE)

#Add Effect Sizes to Data

#------------------------

datSextLongPLO <- escalc(measure = "PLO", xi=ESize, ni=N_SAMPLE, data = datSextLong)

##########################################

## ##

### GLOBAL MEAN PREVALENCES ESTIMATION ###

## ##

##########################################

# =======================================================================================

## Three-level Linear Random-Effects Meta-Analysis using Logit Transformed Proportions ## # =======================================================================================

#Joint Model Fit for All Three Sexting Experiences

#---------------------------------------------------

mv_PLO <- rma.mv(yi, vi, data = datSextLongPLO,

mods = ~ Action - 1, random = ~ Action | STUDY,

struct = "UN", method = meth, slab = paste(datSextLongPLO$STUDY))

#Proportion Estimates for each Sexting Experience, Confidence and Credibility Intervals

#--------------------------------------------------------------------------------------

p_mv <- predict(mv_PLO, transf = transf.ilogit,

newmods = rbind(c(1,0,0),c(0,1,0),c(0,0,1)),

addx = TRUE, digits = dec, tau2.levels = c(1,2,3))

##########################

## ##

### MODERATOR ANALYSIS ###

## ##

##########################

# ==========================================================================================

## Three-level Linear Mixed-Effects Meta-Analysis using Logit Transformed Proportions ##

## ##

## Document Type as an Example of Categorical Moderator ##

# ==========================================================================================

#Joint Model Fit for all three Sexting Experiences

#---------------------------------------------------

mv_PLO_doc <- rma.mv(yi, vi, data = datSextLongPLO,

mods = ~ Action + Action:DOC_TYPE - 1,

random = ~ Action | STUDY,

struct = "UN", method = meth, btt = c(4:6),

tdist=FALSE, slab = paste((datSextLongPLO$STUDY)))

#Proportion Estimates for Each Sexting Experience, Confidence and Credibility Intervals

#--------------------------------------------------------------------------------------

p_mv_Doc <- predict(mv_PLO_doc, transf = transf.ilogit,

newmods=rbind(c(1,0,0,0,0,0),c(1,0,0,1,0,0),

c(0,1,0,0,0,0),c(0,1,0,0,1,0),

c(0,0,1,0,0,0),c(0,0,1,0,0,1)),

addx = TRUE, digits=dec, tau2.levels = c(1,1,2,2,3,3))

# ==========================================================================================

## Three-level Linear Mixed-Effects Meta-Analysis using Logit Transformed Proportions ##

## ##

## Year of data collection as an Example of Quantitative Moderator ##

# ==========================================================================================

#Joint Model Fit for all Three Sexting Experiences

#---------------------------------------------------

mv_PLO_Year <- rma.mv(yi, vi, data = datSextLongPLO,

mods = ~ Action + Action:COL_YEAR - 1,

random = ~ Action | STUDY,

struct = "UN", method=meth, btt=c(4:6),

tdist= FALSE, slab=paste(datSextLongPLO$STUDY))

#Proportion Estimates for each Sexting Experience, Confidence and Credibility Intervals

#--------------------------------------------------------------------------------------

p_mv_Year <- predict(mv_PLO_Year,

newmods=rbind(c(1,0,0,2009,0,0),c(1,0,0,2014,0,0),c(1,0,0,2018,0,0),

c(0,1,0,0,2009,0),c(0,1,0,0,2014,0),c(0,1,0,0,2018,0),

c(0,0,1,0,0,2009),c(0,0,1,0,0,2014),c(0,0,1,0,0,2018)),

transf=transf.ilogit, addx=TRUE, digits=dec,

tau2.levels = c(1,1,1,2,2,2,3,3,3))

#######################################

## ##

### DIAGNOSTIC FUNCTIONS AND GRAPHS ###

## ##

#######################################

#--------------------------------------

#Profile Likehood plots without moderators

#-----------------------------------------

par(mfrow=c(2,4))

profile.rma.mv(mv_PLO, tau2=1)

profile.rma.mv(mv_PLO, tau2=2)

profile.rma.mv(mv_PLO, tau2=3)

| 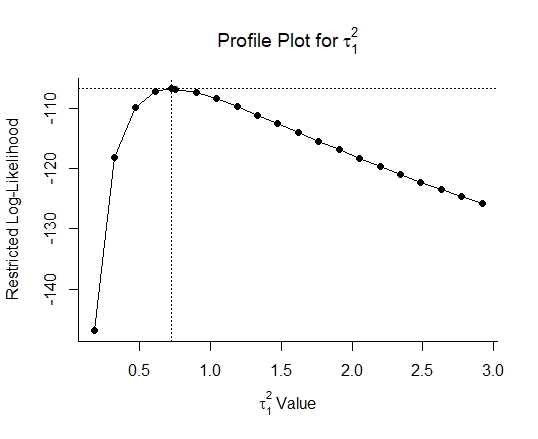 | 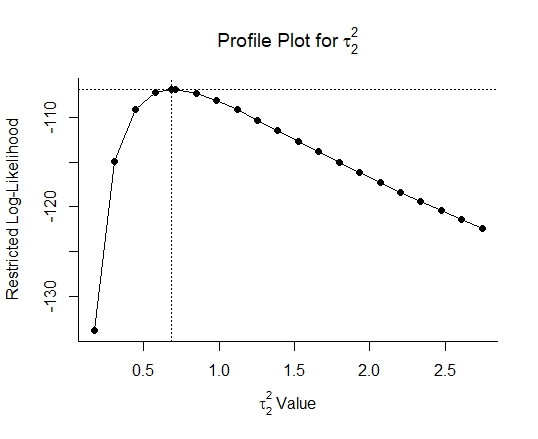 |
| --- | --- |
| 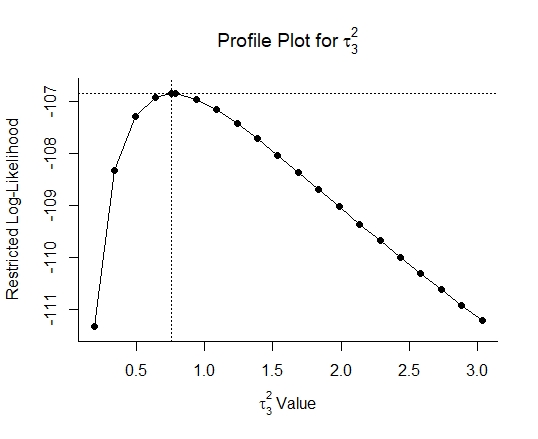 |  |

#Analysis of Potential Influential Studies or Observations

#---------------------------------------------------------

# Note: Studentized Residuals are residuals divided by their estimated standard errors

(like t-statistics).

# Note: Observations with absolute values larger than 3 are considered outliers.

ResStud <- rstudent(mv_PLO)

which( abs(ResStud$z) > 3 )

which( abs(ResStud$z) > 2 )

# Sending: 7-Fix et al. (2019), 56- Maheux et al. (2020);

# Receiving: 65-Gewirtz-Meydan et al. (2018), 72-Mitchell et al. (2012)

boxplot(ResStud$z)

plot(ResStud$z)

| 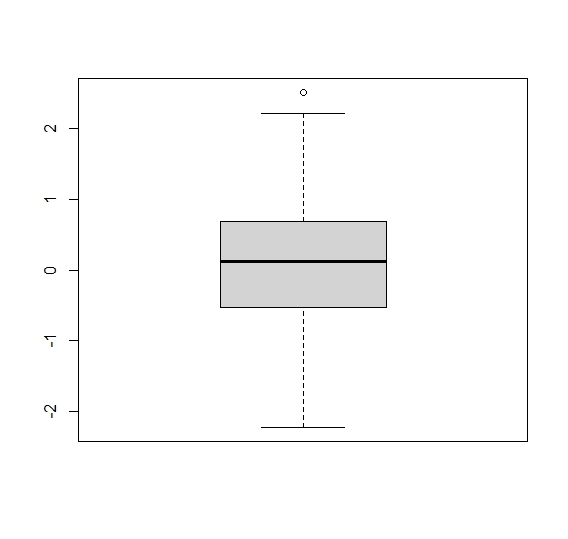 | 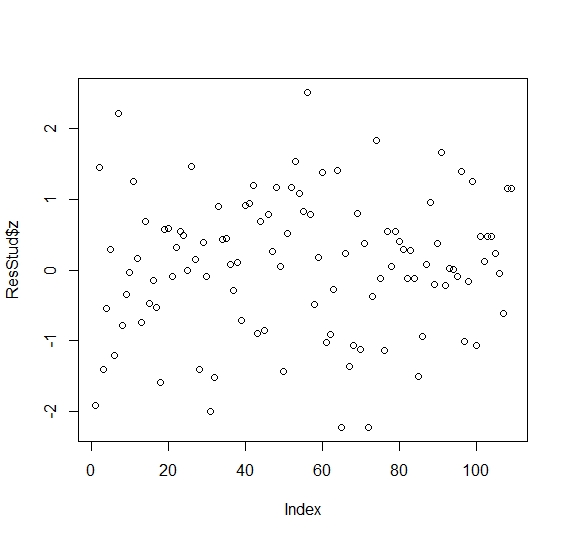 |
| --- | --- |

# Note: Cook's D measure of aggregate impact of each observation on the group of regression

coefficients, as well as the group of fitted values.

# Note: Values larger than 4/n are considered highly influential, where “n” is the sample size.

CookD <- cooks.distance(mv_PLO, transf=transf.ilogit, progbar = TRUE)

which( CookD > (4/109) )

which( CookD > (4/79) )

plot(CookD, type="o", pch=19, xlab="Observed Outcome", ylab="Cook's Distance")


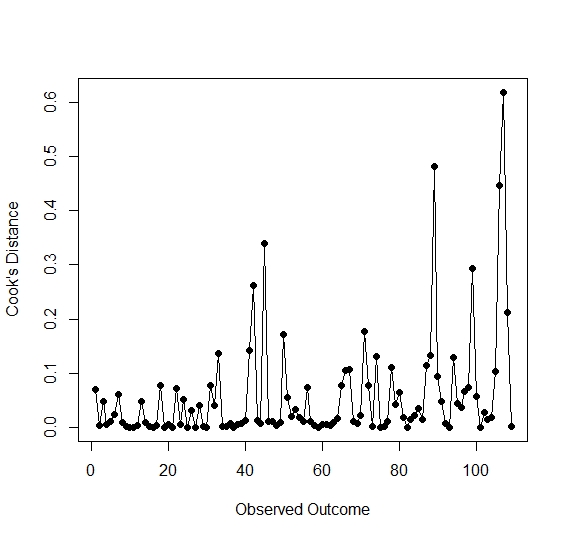


# Note: DFBetas measure of how much an observation has affected the estimate of a regression

coefficient (there is one DFBETA for each regression coefficient).

# Note: Absolute values larger than 2/sqrt(n) are considered highly influential, where “n” is the sample size.

DFBetas <- dfbetas.rma.mv(mv_PLO, transf=transf.ilogit, progbar = TRUE)

which( abs(DFBetas) > (2/sqrt(109)) )

which( abs(DFBetas) > (2/sqrt(79)) )

plot(DFBetas)


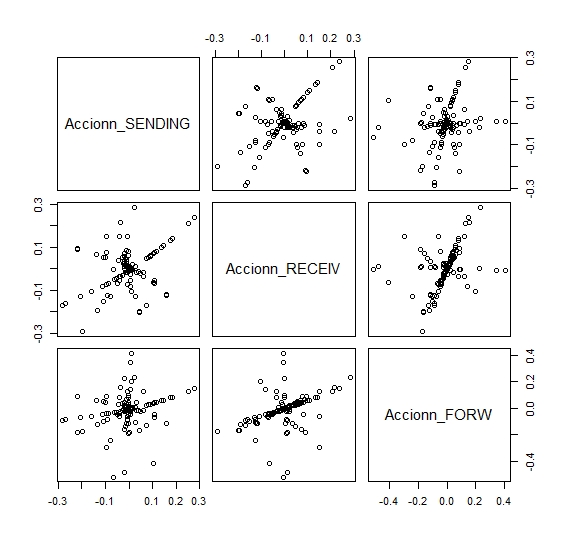


# #Analysis of a Potential Publication Bias

#------------------------------------------

funnel(mv_PLO, back = "white", main="Standard Error", digits = dec)

funnel(mv_PLO, yaxis="vi", back = "white", main="Sampling Variance", digits = dec)

| 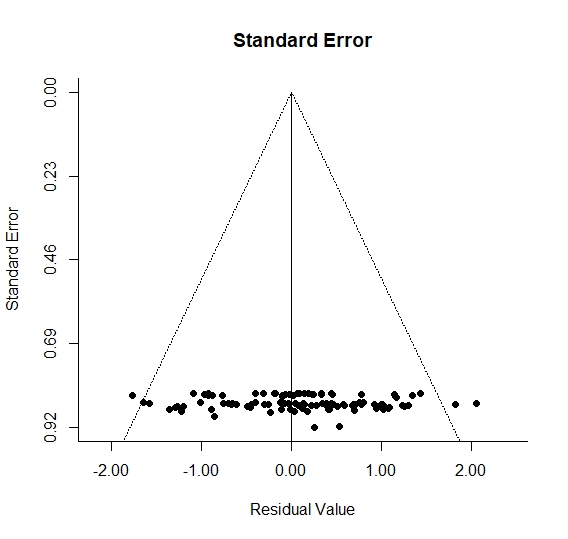 | 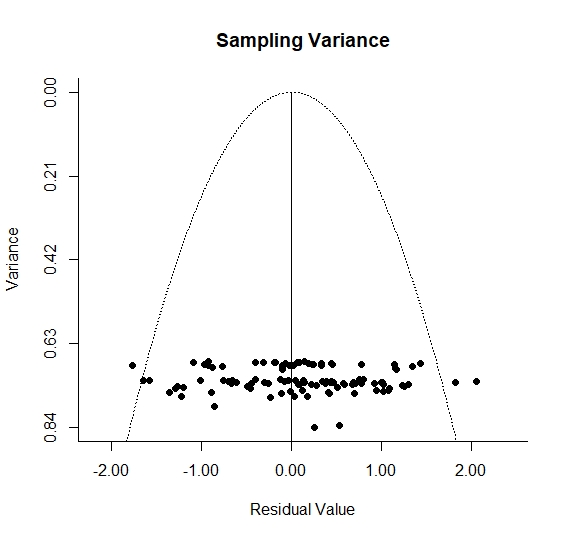 |
| --- | --- |

###################

## ##

### FOREST PLOT ###

## ##

###################

#------------------

#Jointly Forest Plot for Multiple Outcomes

#------------------------------------------

forest(mv_PLO, transf = transf.ilogit, comb.random = TRUE, comb.fixed = FALSE, addfit = TRUE, addcred = 1, showweights = TRUE, overall = TRUE, study.results = TRUE, leftcols = c("studlab"), rightcols = c("effect.ci"), digits = dec, digits.se = dec, digits.zval = dec, digits.tau2 = dec, digits.pval = dec, digits.pval.Q = dec, digits.Q = dec, digits.I2 = dec, digits.weight = dec, digits.mean = dec, digits.sd = dec)

# Note: Forest plots in the article were made using Excel with results from R Code.
