# Supplementary material for: Prevalence of sending, receiving and forwarding sexts among youths: A three-level meta-analysis
Source: PLoS One. 2020 Dec 7;15(12):e0243653. doi: 10.1371/journal.pone.0243653 (PMC7721144; doi:10.1371/journal.pone.0243653)
Supplement: S4 Table — “DT-NPR” = degree thesis no peer-reviewed, “MT-NPR” = master thesis no peer-reviewed, “PT-NPR” = published thesis no peer-reviewed, “R-NPR” = report no peer-reviewed, “A-PR” = article peer-reviewed, “Cs” = cross sectional, “L” = longitudinal, “P” = probabilistic, “NP” = no probabilistic, “Os” = online survey, “Ps” = paper survey, “O&Ps” = online and paper survey, “TI” = telephone interview, “PI” = personal interview, “Mo” = Devices monitorization, “DC” = disaggregated by contents, “na” = not available, insufficient information or unclear. (DOCX) [file pone.0243653.s005.docx]

**S4 A Table.** **Characteristics of included studies.**

|  |  | Document type (Doc), design (De), sampling (Sa) and administration procedure (Admin) | | | |  | n, % women (W) | | Age range: minimum (Mi.), maximum (Ma.) and mean (Me.) | | | % of sending (Send), receiving (Rece) and forwarding (Forw) | | |
| --- | --- | --- | --- | --- | --- | --- | --- | --- | --- | --- | --- | --- | --- | --- |
| N^o^ | Study | Doc | De | Sa | Admin | Country | n | W (%) | Mi. | Ma. | Me. | Send | Rece | Forw |
| 1 | Alfaro-González et al. 2015 | A-PR | Cs | P | O&Ps | Spain | 2412 | 47.30 | 13 | 18 | na | na | 22.80 | na |
| 2 | Arias Cerón et al. 2018 | A-PR | Cs | NP | Os | Chile | 12926 | 62.94 | 5 | 18 | 13.17 | na | 33.80 | na |
| 3 | Baiden et al. 2020 | A-PR | Cs | P | Ps | Rep. of Ghana | 576 | 53 | na | na | 16.10 | 24.70 | na | na |
| 4 | Baumgartner et al. 2014 | A-PR | Cs | P | Ps | Multiple countries | 14946 | 50.30 | 11 | 16 | 13.49 | 3.22 | na | na |
| 5 | Beckmeyer et al. 2019 | A-PR | Cs | P | Os | USA | 600 | 52.05 | 14 | 17 | na | na | na | na |
| 6 | Bermeo 2019 | MT-NPR | Cs | NP | Ps | Ecuador | 259 | 56.80 | 14 | 17 | 15.75 | 35.90 | 58.30 | na |
| 7 | Brinkley et al. 2017 | A-PR | L | NP | Mo | USA | 181 | 47 | 15 | 16 | na | na | na | na |
| 8 | Campbell & Park 2014 | A-PR | Cs | P | TI | USA | 552 | 47.60 | 12 | 17 | 14.88 | 4.50 | 15.60 | na |
| 9 | Chaudhary et al. 2017 | A-PR | L | NP | Os | USA | 500 | 52.40 | na | na | 12.20 | na | na | na |
| 10 | Choi et al. 2019 | A-PR | L | NP | na | USA | 894 | 55.82 | na | na | 17.40 | 20.11 | 29.60 | na |
| 11 | Cleary & Najdowski 2019 | A-PR | Cs | NP | Os | USA | 144 | 47 | 14 | 17 | 15.70 | na | na | na |
| 12 | Cox Communications 2009 | R-NPR | Cs | P | Os | USA | 655 | 49 | 13 | 18 | na | 9 | 17 | 3 |
| 13 | Dawson et al. 2019 | A-PR | Cs | NP | Os | USA | 58 | 27.60 | 13 | 16 | 14.48 | 17.20 | 25.80 | na |
| 14 | De Graaf et al. 2018 | A-PR | Cs | P | Os | Netherlands | 9469 | 60.21 | 12 | 17 | na | 5.50 | na | na |
| 15 | Dodaj et al. 2019 | A-PR | L | NP | Ps | Bosnia & H | 359 | 60.17 | 15 | 17 | 16.32 | 30.92 | 62.95 | na |
| 16 | Dolev-Cohen & Ricon 2020 | A-PR | Cs | NP | Os | Israel | 458 | 77.95 | 12 | 18 | 15.57 | 28.80 | na | na |
| *Continued* | |  |  |  |  |  |  |  |  |  |  |  |  |  |

| N^o^ | Study | Doc | De | Sa | Admin | Country | n | W (%) | Mi. | Ma. | Me. | Send | Rece | Forw |
| --- | --- | --- | --- | --- | --- | --- | --- | --- | --- | --- | --- | --- | --- | --- |
| 17 | Fix et al. 2019 | A-PR | Cs | NP | PI | USA | 561 | 0 | na | na | na | 49.91 | na | na |
| 18 | Frankel et al. 2018 | A-PR | Cs | P | Ps | USA | 6021 | 49.40 | na | na | na | na | na | na |
| 19 | Gámez-G. & Mateos-Pérez 2019 | A-PR | L | P | Ps | Spain | 1497 | 53.20 | 12 | 14 | 13.65 | 7.60 | na | na |
| 20 | Gámez-G. & Santisteban 2018 | A-PR | L | P | Ps | Spain | 1208 | 52.81 | 12 | 16 | 13.57 | 10.70 | na | na |
| 21 | Gámez-Guadix et al. 2017 | A-PR | Cs | P | Ps | Spain | 3223 | 49.90 | 12 | 17 | 14.06 | 13.50 | na | na |
| 22 | Garitaonandia et al. 2019 | A-PR | Cs | P | PI | Spain | 500 | na | 9 | 16 | na | na | 31 | na |
| 23 | Gerding 2016 | PT-NPR | Cs | NP | Ps | USA | 201 | 53.23 | 14 | 17 | 16.01 | 32.30 | 58.70 | na |
| 24 | Gewirtz-Meydan et al. 2018 | A-PR | Cs | P | TI | USA | 1560 | 50.30 | 10 | 17 | 14.20 | na | 7.05 | na |
| 25 | Ghorashi et al. 2019 | A-PR | Cs | P | Ps | Iran | 944 | 60.59 | 15 | 18 | 15.48 | 15.70 | 34.70 | 6.30 |
| 26 | Gregg et al. 2018 | A-PR | Cs | NP | Ps | USA | 314 | 51.27 | na | na | 16.20 | DC | DC | na |
| 27 | Gutiérrez-Gómez 2019 | DT-NPR | Cs | NP | Os | Mexico | 530 | 55.85 | 12 | 16 | na | 4.53 | 30.19 | na |
| 28 | Hinduja & Patchin 2010 | R-NPR | Cs | P | na | USA | 4365 | 49.50 | 11 | 18 | na | 7.70 | 12.90 | na |
| 29 | Houck et al. 2014 | A-PR | Cs | NP | Os | USA | 410 | na | 12 | 14 | na | 22.43 | na | na |
| 30 | Kim et al. 2019 | A-PR | Cs | P | Os | Canada | 2537 | 48.40 | 14 | 17 | 15.42 | 14.40 | 27 | na |
| 31 | Kopecký 2012 | A-PR | Cs | NP | Os | Czech Republic | 9353 | 47.41 | 11 | 17 | na | 9.70 | na | na |
| 32 | Kopecký 2014 | A-PR | Cs | NP | Os | Czech Republic | 21372 | 55.42 | 11 | 17 | na | 12.42 | na | na |
| 33 | Kopecký 2015 | A-PR | Cs | NP | Os | Czech Republic | 1237 | na | 11 | 17 | na | 9.31 | na | na |
| 34 | Lenhart 2009 | R-NPR | Cs | P | TI | USA | 800 | 48.70 | 12 | 17 | na | 4 | 15 | na |
| 35 | León-Prieto et al. 2017 | A-PR | Cs | NP | Ps | Ecuador | 205 | 61.95 | 15 | 17 | na | na | na | na |
| 36 | Lippman & Campbell 2014 | A-PR | Cs | NP | Ps | USA | 51 | 49 | 12 | 18 | 14.55 | 21 | 48 | na |
| 37 | Livingstone & Görzig 2014 | A-PR | Cs | P | O&Ps | Multiple countries | 15619 | na | 11 | 16 | na | na | 15 | na |
| *Continued* | |  |  |  |  |  |  |  |  |  |  |  |  |  |

| N^o^ | Study | Doc | De | Sa | Admin | Country | n | W (%) | Mi. | Ma. | Me. | Send | Rece | Forw |
| --- | --- | --- | --- | --- | --- | --- | --- | --- | --- | --- | --- | --- | --- | --- |
| 38 | Lucić et al. 2020 | A-PR | Cs | NP | Os | Croatia | 319 | 66.10 | na | na | 16.50 | 36.99 | na | na |
| 39 | Maas et al. 2018 | A-PR | Cs | NP | Os | USA | 312 | 100 | na | na | 15.21 | 21.20 | na | na |
| 40 | Maheux et al. 2020 | A-PR | Cs | NP | Os | USA | 626 | 53.50 | na | na | 17.40 | 55.60 | na | na |
| 41 | Marcum et al. 2014 | A-PR | Cs | NP | Ps | USA | 1617 | 49.70 | na | na | 15.77 | 13 | na | na |
| 42 | Medina & Verdugo 2018 | DT-NPR | Cs | NP | Ps | Ecuador | 463 | 41 | 15 | 18 | 16.04 | 17.50 | 38 | 17.10 |
| 43 | Mishna et al. 2010 | A-PR | Cs | P | Ps | Canada | 2186 | 54.70 | na | na | na | na | na | 3 |
| 44 | Mitchell et al. 2012 | A-PR | Cs | P | TI | USA | 1560 | 50 | 10 | 17 | 14.20 | na | 7.10 | na |
| 45 | Molla-Esparza et al. 2020 | A-PR | Cs | NP | Ps | Spain | 647 | 47.80 | 12 | 18 | 13.70 | 24.27 | 57.96 | 17.93 |
| 46 | Montiel et al. 2016 | A-PR | Cs | P | Ps | Spain | 3897 | 52.58 | 12 | 17 | 14.45 | na | 24.40 | na |
| 47 | Naezer 2018 | A-PR | Cs | NP | Os | Netherlands | 679 | na | 12 | 18 | na | 20.32 | na | na |
| 48 | Nielsen et al. 2015 | A-PR | Cs | NP | Os | Finland | 1296 | 100 | 11 | 18 | na | 20 | 65 | na |
| 49 | O'Sullivan 2014 | A-PR | Cs | NP | Os | Canada | 269 | 66 | 13 | 17 | 17 | 13.80 | 28.60 | na |
| 50 | Patrick et al. 2015 | A-PR | Cs | NP | O&Ps | Australia | 2114 | 61.64 | na | na | na | DC | DC | na |
| 51 | Quesada et al. 2018 | A-PR | Cs | NP | O&Ps | Spain | 303 | 51.70 | 14 | 18 | 15.35 | 35.60 | na | na |
| 52 | Rice et al. 2012 | A-PR | Cs | P | Ps | USA | 1839 | 48.14 | 12 | 18 | 15.32 | 15.44 | na | na |
| 53 | Rice et al. 2014 | A-PR | Cs | P | Ps | USA | 1173 | 48.48 | 10 | 15 | 12.30 | 4.60 | 20.10 | na |
| 54 | Rice et al. 2018 | A-PR | Cs | P | Ps | USA | 1208 | 51.50 | 12 | 18 | 16 | 18.50 | 40.90 | na |
| 55 | Ricketts et al. 2015 | A-PR | Cs | NP | Ps | USA | 1617 | 51 | na | 18 | 15.77 | 13 | na | na |
| 56 | Ševčíková 2016 | A-PR | Cs | P | Ps | Multiple countries | 17016 | 50.30 | 11 | 16 | 13.55 | 3 | na | na |
| 57 | Ševčíková et al. 2018 | A-PR | L | NP | Os | Czech Republic | 1134 | 58.80 | 10 | 18 | 13.84 | 4.20 | na | na |
| 58 | Soriano et al. 2019 | A-PR | Cs | P | na | Spain | 603 | 51.08 | 12 | 17 | 14.43 | 16.75 | 38.14 | 16.09 |
| *Continued* | |  |  |  |  |  |  |  |  |  |  |  |  |  |

| N^o^ | Study | Doc | De | Sa | Admin | Country | n | W (%) | Mi. | Ma. | Me. | Send | Rece | Forw |
| --- | --- | --- | --- | --- | --- | --- | --- | --- | --- | --- | --- | --- | --- | --- |
| 59 | Stanley et al. 2018 | A-PR | Cs | NP | Ps | Multiple countries | 4564 | na | 14 | 17 | na | 25.57 | 32.04 | na |
| 60 | Steinberg et al. 2019 | A-PR | L | NP | Ps | USA | 429 | 54 | na | na | na | 24 | na | na |
| 61 | Strassberg et al. 2013 | A-PR | Cs | NP | Ps | USA | 606 | 45.87 | na | na | na | 20 | 41 | 10.23 |
| 62 | Strassberg et al. 2014 | A-PR | Cs | NP | Ps | USA | 1130 | 59.20 | na | na | na | 19.10 | 38.20 | 7.80 |
| 63 | Strassberg et al. 2017 | A-PR | Cs | NP | Ps | USA | 622 | 45.99 | na | na | na | 14.79 | 36.01 | 10.13 |
| 64 | Titchen et al. 2019 | A-PR | Cs | NP | na | USA | 555 | 62.70 | 14 | 17 | 15.60 | 22.34 | na | na |
| 65 | V. Ouytsel et al. 2019a | A-PR | Cs | NP | Ps | Belgium | 3109 | 53.50 | 12 | 15 | 13.01 | 7.40 | 26.70 | 4.50 |
| 66 | V. Ouytsel et al. 2019b | A-PR | Cs | NP | Ps | Belgium | 657 | 63.30 | 17 | 18 | 16.41 | 30.30 | na | na |
| 67 | V. Ouytsel, Ponnet et al. 2014 | A-PR | Cs | NP | Ps | Belgium | 329 | 60.20 | 15 | 18 | 16.71 | DC | 28.70 | na |
| 68 | V. Ouytsel, Van Gool et al. 2014 | A-PR | Cs | NP | Ps | Belgium | 1028 | 58 | 15 | 18 | 16.68 | 11.10 | na | na |
| 69 | Vanden Abeele et al. 2012 | A-PR | Cs | NP | Ps | Belgium | 540 | 57 | na | na | 16.07 | na | na | na |
| 70 | Velarde 2014 | DT-NPR | Cs | NP | Ps | USA | 661 | 46 | na | na | na | 15 | 35.80 | 10.20 |
| 71 | Villacampa 2016 | A-PR | Cs | NP | Ps | Spain | 489 | 50.10 | 14 | 18 | 15.64 | na | 28.60 | 8.20 |
| 72 | Villanueva & Serrano 2019 | A-PR | Cs | NP | Ps | Spain | 163 | 41.70 | 12 | 16 | 13.63 | na | 11 | na |
| 73 | Wachs et al. 2017 | A-PR | Cs | NP | O&Ps | Multiple countries | 1818 | 52.50 | 12 | 17 | 14.32 | 8 | na | na |
| 74 | Walrave et al. 2014 | A-PR | Cs | NP | Ps | Belgium | 498 | 54 | 15 | 18 | na | 26 | na | na |
| 75 | West et al. 2014 | A-PR | Cs | NP | Ps | Peru | 949 | 65.65 | 12 | 18 | 14.75 | na | na | na |
| 76 | Wolfe et al. 2016 | A-PR | Cs | P | TI | USA | 625 | na | 12 | 17 | na | na | 17 | na |
| 77 | Wood et al. 2015 | A-PR | Cs | NP | Ps | Multiple countries | 3277 | 47.33 | 14 | 17 | 15 | 26.25 | 33.62 | na |
| 78 | Woodward et al. 2017 | A-PR | Cs | NP | Ps | USA | 548 | 58.21 | na | na | 15.90 | 30.71 | 48.90 | 7.12 |
| 79 | Ybarra & Michell 2014 | A-PR | Cs | P | Os | USA | 3715 | 56.58 | 13 | 18 | na | 7 | na | na |

Table legend. “DT-NPR” = degree thesis no peer-reviewed, “MT-NPR” = master thesis no peer-reviewed, “PT-NPR” = published thesis no peer-reviewed, “R-NPR” = report no peer-reviewed, “A-PR” = article peer-reviewed, “Cs” = cross sectional, “L” = longitudinal, “P” = probabilistic, “NP” = no probabilistic, “Os” = online survey, “Ps” = paper survey, “O&Ps” = online and paper survey, “TI” = telephone interview, “PI” = personal interview, “Mo” = Devices monitorization, “DC” = disaggregated by contents, “na” = not available, insufficient information or unclear.

S4 B Table. Geographical origin of the samples of the studies included in the review.

|  | Sexting experiences  (*k*, %) | | | | | |
| --- | --- | --- | --- | --- | --- | --- |
| Geographical region and countries | Sending  (57, 100%) | | Receiving  (39, 100%) | | Forwarding  (13, 100%) | |
| Europe | 23 | 40.35% | 14 | 35.90% | 4 | 30.77% |
| Netherlands | 2 | 3.51% |  |  |  |  |
| Bosnia & H. | 1 | 1.75% | 1 | 2.56% |  |  |
| Spain | 6 | 10.53% | 7 | 17.95% | 3 | 23.08% |
| Czech Republic | 4 | 7.02% |  |  |  |  |
| Croatia | 1 | 1.75% |  |  |  |  |
| Finland | 1 | 1.75% | 1 | 2.56% |  |  |
| Belgium | 4 | 7.02% | 2 | 5.13% | 1 | 7.69% |
| Multiple countries | 4 | 7.02% | 3 | 7.69% |  |  |
| North America | 28 | 49.12% | 21 | 53.85% | 7 | 53.85% |
| Canada | 2 | 3.51% | 2 | 5.13% | 1 | 7.69% |
| Northern America | 25 | 43.86% | 18 | 46.15% | 6 | 46.15% |
| Mexico | 1 | 1.75% | 1 | 2.56% |  |  |
| South America | 2 | 3.51% | 3 | 7.69% | 1 | 7.69% |
| Chile |  |  | 1 | 2.56% |  |  |
| Ecuador | 2 | 3.51% | 2 | 5.13% | 1 | 7.69% |
| Africa | 1 | 1.75% |  |  |  |  |
| Rep. of Ghana | 1 | 1.75% |  |  |  |  |
| Asia | 2 | 3.51% | 1 | 2.56% | 1 | 7.69% |
| Israel | 1 | 1.75% |  |  |  |  |
| Iran | 1 | 1.75% | 1 | 2.56% | 1 | 7.69% |
| Not Applicable | 1 | 1.75% |  |  |  |  |

Table legend. “k” = number of studies.
